# Toward Prostate Cancer Early Warning with a Self‐Powered Wearable Biosensing Platform Integrated with Machine Learning

**DOI:** 10.1002/advs.202524234

**Published:** 2026-02-27

**Authors:** Jing Xu, Hanxiao Chen, Qichen Yuan, Liucun Yin, Yifang Tao, Hong Wang, Yuquan Xue, Delai Fu, Huan Pang, Tie Chong, Li Xue

**Affiliations:** ^1^ Department of Urology, The Second Affiliated Hospital of Xi'an Jiaotong University Xi'an 710004 China; ^2^ College of Chemistry and Chemical Engineering Xinyang Normal University Xinyang 464000 China; ^3^ School of Chemistry and Chemical Engineering Yangzhou University Yangzhou 225009 China

**Keywords:** hybrid‐powered chip, sarcosine detection, urine‐triggered, wearable chips

## Abstract

Current prostate cancer detection methods remain limited in non‐invasiveness and specificity, prompting interest in urinary biomarkers such as sarcosine. Here, we report a urine‐powered wearable platform for non‐invasive sarcosine detection as a proof‐of‐concept for decentralized early warning. Distinct from prior urine‐powered sensors, this platform establishes a hybrid bio‐electrochemical energy paradigm in which urine simultaneously functions as the analytical sample and an intrinsic energy reservoir. Specifically, sarcosine serves as the biofuel to drive an enzymatic fuel cell, while endogenous Zn^2^^+^ ions in urine power a complementary aqueous battery component, enabling synergistic signal amplification and stable operation. This synergistic design enables strong signal amplification and operational stability, achieving a detection limit as low as 0.85 pm. Dual electrochemical and colorimetric readouts allow flexible result interpretation. A hybrid classification‐regression model is implemented to identify high‐concentration samples using a learned threshold, followed by range‐specific regression to reduce dynamic‐range interference and improve quantitative accuracy. The system is further integrated into a smart diaper format with wireless connectivity for on‐demand smartphone‐based analysis. Preliminary validation using human urine samples shows satisfactory analytical performance, with spike‐and‐recovery values ranging from 83.68% to 125.88%. This work provides a proof‐of‐concept demonstration of a sarcosine‐centered, self‐powered sensing strategy for non‐invasive biomarker detection.

## Graphical abstract

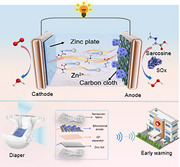

## 1 Introduction

Prostate cancer ranks among the most prevalent malignancies in men worldwide, making early diagnosis crucial for improving patient outcomes [1, 2]. Current clinical reliance on prostate‐specific antigen (PSA) testing faces limitations due to insufficient specificity, frequently leading to false‐positive results, unnecessary biopsy procedures, and overdiagnosis of indolent tumors [3, 4]. These shortcomings underscore the urgent need to identify more reliable, non‐invasive biomarkers. Urine, as an easily accessible biological sample, offers unique advantages in cancer diagnosis, including non‐invasive collection, a rich source of biomarkers, and the ability to reflect dynamic metabolic information. Notably, urine directly contains prostate secretions, which can reveal the pathological state of the prostate. Among various potential biomarkers, sarcosine (SAR) has garnered significant attention due to its close association with prostate cancer progression [5, 6]. Elevated SAR levels are strongly linked to tumor aggressiveness and metastatic potential, making it an ideal target for early diagnosis. However, detecting SAR in urine faces challenges such as low concentration and a complex matrix. Traditional methods like chromatography and mass spectrometry are costly and difficult to adapt for point‐of‐care testing [7, 8, 9].

Enzyme biofuel cell (EBFC)‐based self‐powered sensor exhibit great potential in wearable medical detection due to their advantages such as external power‐free operation and environmental friendliness [10, 11, 12, 13, 14]. The sensor directly converts chemical energy into electricity through enzymatic catalytic reactions, enabling self‐powered detection of target analytes. Particularly for urinary biomarker detection, EBFC systems can effectively utilize endogenous substances in urine as fuel to provide continuous energy for the sensing process. For example, the Shi research group constructed a paper‐based biofuel cell (BFC) using urinary glucose as fuel. They integrated it with a wireless transmission device to create a self‐powered diaper sensor. This system achieved urine glucose detection in the range of 0–10 mm, providing a new method for diabetes monitoring [15]. Although performance enhancements of EBFC have been achieved to some extent through strategies such as nanomaterial‐modified electrodes and enzyme immobilization techniques [16, 17, 18]. These improvements offer limited signal amplification effects and fail to fundamentally address their core limitation of insufficient sensitivity. This is primarily due to the inherent kinetic constraints of enzymatic reactions, the intrinsic trade‐off between biocompatibility and stability, and interference from complex biological matrices on electron transfer efficiency [19, 20]. Particularly for early‐stage prostate cancer screening, the extremely low concentration of SAR in patient urine makes reliable detection challenging for conventional EBFC systems. It is noteworthy that the rapid development of energy storage technology has provided new solutions for wearable sensing systems. He et al. developed a wearable device integrating energy storage and sensing functions. A solid‐state ZIB provides a reliable power supply, while a highly sensitive pressure sensor enables monitoring of pulse and respiration. This integration achieves self‐powering capability and high‐sensitivity detection, demonstrating promising potential for intelligent medical monitoring applications [21]. Among these new energy storage systems, ZIB are particularly suitable for urine testing due to their unique performance advantages [22, 23]. First, compared to lithium/sodium‐ion batteries with organic electrolytes, ZIB use aqueous electrolytes, which can offer high safety and exhibit excellent compatibility with biological samples. Second, zinc electrodes demonstrate excellent stability in physiological environments and are less prone to oxidation side reactions. More importantly, the naturally occurring zinc ions in urine can directly serve as an electrolyte source, significantly simplifying system design [24]. However, the development of ZIB is still constrained by the challenge of balancing structural stability and ion diffusion kinetics in cathode materials. Traditional layered materials are prone to structural collapse during cycling, while transition metal chalcogenides exhibit potential advantages due to their unique electronic structures and multi‐electron reaction characteristics [25, 26]. Among various candidate materials, cubic‐phase CuSe has attracted attention for its open crystal framework and high theoretical capacity, yet its bulk form still faces issues such as low active‐site utilization and significant volume change [27, 28]. Inspired by the ability of hollow structures to buffer volume strain and facilitate ion transport. Constructing a porous framework through a synergistic strategy of geometric structure innovation could provide continuous charge transport pathways while exposing abundant active interfaces [29]. This approach offers a new perspective for overcoming the capacity‐stability trade‐off in ZIB cathode materials.

Therefore, this work proposes an innovative approach by integrating a high‐power ZIB with a sarcosine‐fueled EBFC to construct a hybrid‐power bio‐sensing system. The design leverages the ZIB to provide stable, high‐power output while utilizing the EBFC for target‐specific recognition, thereby overcoming the limitations of conventional self‐powered sensors in terms of insufficient sensitivity and poor specificity. Additionally, a custom‐designed Bluetooth transmission module enables real‐time data synchronization to a smartphone app, facilitating intelligent monitoring of prostate cancer biomarkers. By utilizing the byproducts of electrochemical reactions to trigger the in situ colorimetric reaction of electrochromic materials, the electrochemical‐colorimetric output system was contrasted. This design preserves the high sensitivity of electrochemical detection and provides an intuitive visual readout capability, effectively addressing the applicability challenges of portable detection in complex environments. By synergistically integrating ZIB power supply, EBFC recognition, and dual‐mode signal conversion, this work achieves a groundbreaking innovation spanning energy provision, biological detection, and signal output, overcoming the multiple limitations of conventional self‐powered sensors in reliability, universality, and user experience. The Bluetooth‐enabled smartphone interaction further provides a new technical paradigm for the design of high‐sensitivity, intelligent point‐of‐care biosensing systems, providing an entirely new design logic and technical pathway for developing point‐of‐care testing (POCT) devices.

## 2 Experimental

### 2.1 Zinc‐Ion Battery Structure

The N‐methylpyrrolidone (NMP) slurry comprising the CuSe cathode material, conductive carbon black and 10 wt.% polyvinylidene fluoride (PVDF) binder were mixed together in a rational proportion and ground to give a homogenous slurry before coating onto aluminium foil current collector. The coated electrode was vacuum‐dried at 80°C for 10 h in order to manufacture the final working electrode. The dried electrodes were punched out to circular discs of 10 mm diameter. For electrochemical performance tests, CR2032‐type coin cells were assembled using an aqueous ZnSO_4_ solution as the electrolyte and zinc foil as the counter for the working electrode.

### 2.2 Sensor Construction

Carbon black was first oxidized in nitric acid at 90°C for 6 h to introduce carboxyl groups on its surface, thereby facilitating subsequent covalent binding with the enzyme. The modified carbon black was then mixed with CuSe and PVDF in NMP to form a uniform suspension. Subsequently, 50 µL of this suspension was drop‐cast onto a piece of carbon cloth, followed by vacuum drying to remove the NMP, thus preparing the base electrode. Next, 30 µL of an EDC/NHS‐containing buffer solution was added onto the electrode surface for activation for 30 min to enable covalent coupling between the enzyme and the electrode. Afterward, 30 µL of SOx (5 mg/mL) was added to the electrode and incubated overnight at 4°C to form a sensing layer capable of responding to the enzymatic reaction. SOx was covalently immobilized on the electrode via EDC/NHS coupling, with an enzyme loading of ∼150 µg per 1 × 1 cm^2^ electrode (≈ 4.5 U per electrode). This covalent immobilization strategy minimizes enzyme leaching and ensures sufficient catalytic activity under electrochemical working conditions. In this way, the anode was obtained. A thin zinc sheet (1 × 1 cm^2^) was used as the cathode.

### 2.3 Ethics Statement

All human urine samples used in this study were collected in accordance with ethical requirements and strictly followed relevant medical guidelines. All procedures for sample collection and use were approved by the Medical Ethics Committee of the Second Affiliated Hospital of Xi'an Jiaotong University (Approval No. 2025YS381). All participants provided informed consent for the use of their urine samples for scientific research.

## 3 Results and Discussion

### 3.1 Sensing Chips Detection Mechanism

**SCHEME 1:**
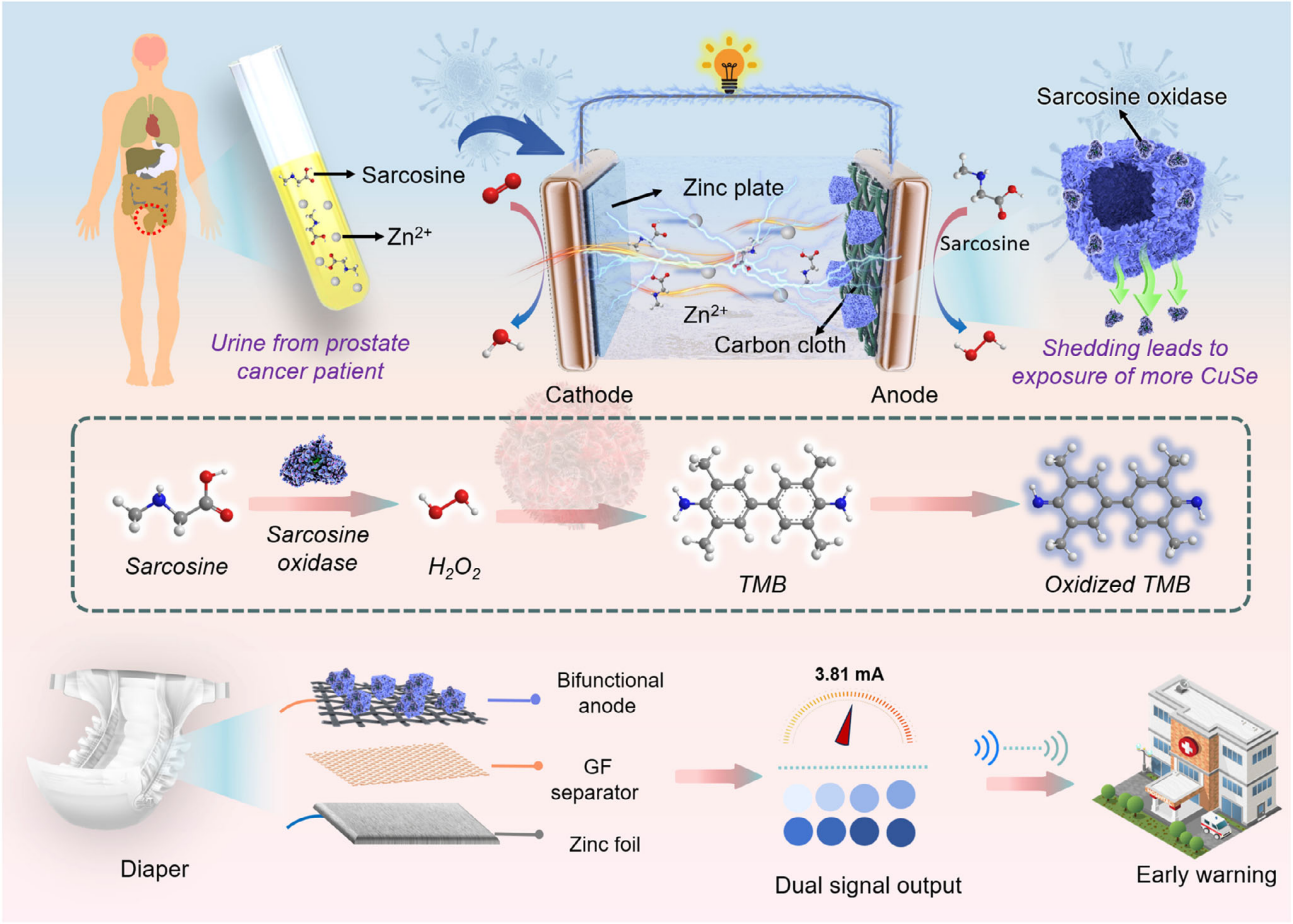
Schematic diagram of the innovative non‐invasive prostate cancer screening system.

Scheme 1 illustrates an innovative non‐invasive prostate cancer screening system that achieves dual‐mode highly sensitive detection through a urine self‐powered hybrid energy supply approach. In the specific detection module, sarcosine oxidase modified on the surface of hollow cube electrodes selectively identifies SAR metabolites in the urine of prostate cancer patients. Zinc ions in the urine serve as the electrolyte for the ZIB, while SAR acts as the fuel for the EBFC, fundamentally enhancing detection sensitivity through dual energy supply. Simultaneously, by‐products of the electrochemical reaction trigger a visible color change in the nonwoven colorimetric strip embedded in the urine collection pad, providing intuitive qualitative assessment. Finally, a wireless transmission module sends the detection data in real time via Bluetooth to smartphones and hospital monitoring systems for quantitative analysis. Although operating simultaneously, the EBFC and ZIB function through distinct, spatially confined mechanisms. The EBFC is controlled by enzyme–substrate specificity and H_2_O_2_‐mediated electron transfer, whereas the ZIB is dominated by Zn^2+^ transport and reversible electrochemical reactions. Consequently, urine matrix variations affect the two subsystems through orthogonal pathways, enabling signal enhancement without mutual interference. This system innovatively integrates highly specific biosensing, self‐powered technology, and wearable devices, enabling both rapid at‐home screening through color changes and precise clinical diagnosis via electronic data. It effectively addresses the issue of high false‐positive rates associated with traditional PSA testing.

### 3.2 Material Morphology

**FIGURE 1:**
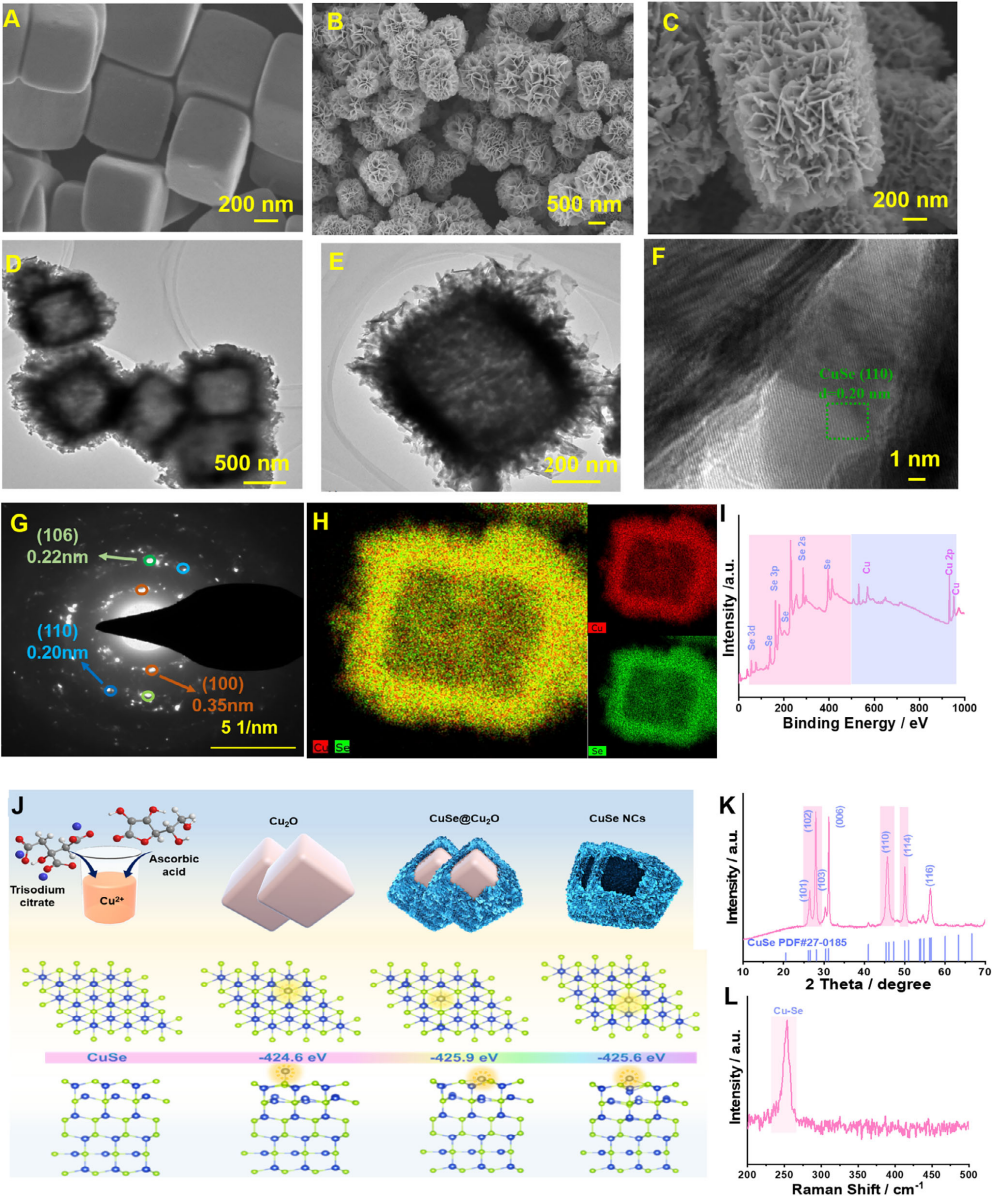
(A) SEM images of Cu_2_O template, (B, C) SEM image of prepared CuSe, (D, E) TEM images of CuSe, (F) High‐resolution TEM (HRTEM) of CuSe, (G) SAED of CuSe, (H) mapping images of CuSe before, (I) XPS spectra of CuSe, (J) Schematic illustration of CuSe synthesis and ball‐and‐stick models of the CuSe material viewed from different angles, along with the three possible adsorption sites on the CuSe surface, (K) X‐ray diffraction (XRD) pattern of CuSe, (L) Raman spectrum of CuSe.

Figure 1A shows the SEM images of the prepared Cu_2_O cube template, which exhibits a regular cubic morphology with a uniform size of ∼500 nm and smooth surfaces without obvious agglomeration. The corresponding TEM images (Figure S1) further confirms the well‐defined nanostructure and clear lattice fringes of the Cu_2_O cubes, indicating good crystallinity. Figure 1B,C shows the SEM image of the prepared CuSe material. Compared with It can be found that the overall morphology remains unchanged after Se processing, and there is still no obvious agglomeration of the basic particles. Based on retaining the original cubic form, the target product is composed of ultra‐thin 2D nano‐sheets with a uniformly rough surface of the nano‐cube. As shown in Figure 1D,E, the hollow CuSe is obvious and the diameter is about 600 nm. Hollow microstructure enables the nanomaterials to have low mass density and avoid the caking phenomenon. The introduction of 2D nanosheets can avoid the fragmentation of 3D thin layer structure and facilitate the infiltration of electrolytes, thus exposing rich active sites and providing ion diffusion pathways. In Figure 1F, lattice fringes with a spacing of 0.20 nm were observed by edge high‐resolution TEM (HRTEM) which is related to the (110) crystal face of CuSe. The selected area electron diffraction pattern (SAED, Figure 1G) shows the single crystal spot and polycrystal diffraction ring corresponding to the (106) / (100) / (110) plane which demonstrates the successful synthesis of polycrystalline CuSe. The mapping image represents the entire nanocube, and the uniform distribution of Cu and Se elements also confirms the successful preparation of composite multidimensional materials (Figure 1H). Element analysis together with SEM observations reveals that the hollow cubic structure of CuSe undergoes partial fragmentation after cycling, accompanied by the emergence of new elemental signals (Figures S2 and S3), indicating that the material undergoes certain structural and compositional evolution during the charge–discharge process. The presence of zinc further verified the successful progression of the reversible reaction. The distribution of EDX elements (Figure S4) and the full spectrum of XPS (Figure 1I) confirms the existence of the main elements of Cu and Se in the material surface, and the above results confirmed the successful preparation of the CuSe. Figure S5 shows two strong characteristic peaks corresponding to Cu 2p_3/2_ (931.7 eV) and Cu 2p_1/2_ (951.6 eV), indicating the presence of the chemical state of Cu^2+^. Figure 1J depicts the preparation procedure of CuSe materials, together with ball‐and‐stick models showing various perspectives and three candidate adsorption sites. Figures S6 and 1K shows the XRD patterns of Cu_2_O and CuSe crystals, which match well with the standard (PDF#05‐0667 and PDF#27‐0185), confirming their phase purity. Moreover, the observed sharp peaks and almost no miscellaneous peaks confirmed the successful synthesis of CuSe. Figure 1L displays the Raman date, and the most intense peak in the spectrum situated at 260 cm^−1^ cans be assigned to the first longitudinal optic phonon mode of Cu‐Se vibrations. These findings further verify the successful synthesis of CuSe.

### 3.3 Discharge Performance and Mechanism Analysis

**FIGURE 2:**
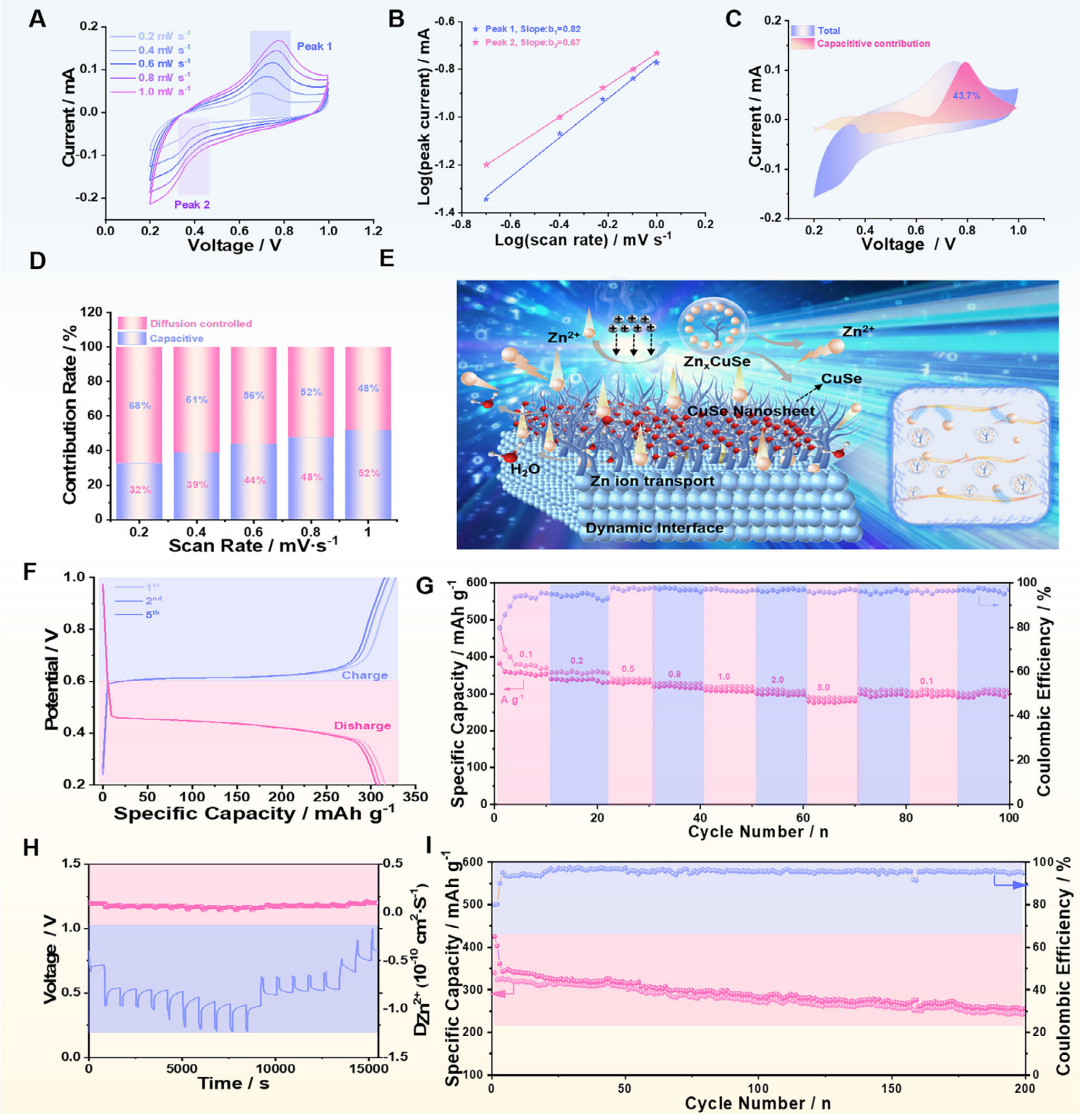
(A) CV curves at various scan rates were recorded in a neutral 2 m Zn^2+^ electrolyte, (B) Log (i) vs log (v) curves of two peaks, (C, D) Capacity contribution rate, (E) Schematic diagram of the constructed ZIB, (F) Charge–discharge curves at the initial five cycles, (G) Rate capability, (H) Discharge–charge GITT profiles, (I) Cycle performance.

The Zn^2+^ diffusion kinetics were investigated by cyclic voltammetry (CV) at different scan rates based on the Randles–Sevcik relationship, using a 2 m Zn^2+^ electrolyte under neutral pH conditions. Well‐defined redox peaks and corresponding discharge/charge plateaus at approximately 0.60 and 0.40 V (vs Zn^2+^/Zn) indicate the reversible Zn^2+^ intercalation/deintercalation behavior of the CuSe electrode. As shown in Figure 2A, the overall CV profiles remain nearly unchanged with increasing scan rate, suggesting low polarization and favorable rate capability. Figure 2B presents the linear fitting of peak current versus scan rate. The *b*‐values of the oxidation and reduction peaks are 0.67 and 0.82, respectively, indicating that Zn^2^^+^ storage in the CuSe cathode is jointly governed by ion diffusion and surface pseudocapacitive effects. The respective contributions of these two processes at different scan rates were further quantified using the same model. Figure 2C shows the CV curve at 0.6 mV s^−1^, where the capacity contribution of pseudo capacitance behavior is 43.7% (purple area). In Figure 2D, the capacity contribution of the pseudo capacitance behavior gradually increases from 32% to 52%. The above analysis can result in a high Zn^2+^ diffusivity, where the empty structure contributes to the material's multi‐form energy storage. Figure S7 shows the electrochemical impedance spectra of the assembled battery at different cycle numbers. The nearly unchanged semicircle size and curve profile after 10 cycles indicate stable interfacial charge transfer and smooth Zn^2+^ diffusion during reversible cycling, confirming the good cycling stability of the battery. Figure S7B displays the linearity correlation between Re (Z) and ω^−1/2^. After the tenth cycle test, the impedance is slightly increased, and the σ fitted through plotting Z' vs ω^−1/2^ in the low‐frequency region is increased from 50 to 60 Ω s^−1/2^, which lead to a slight decrease in the diffusion coefficient. Figure 2E illustrates the intercalation and deintercalation of zinc ions in the electrode material and the catalytic reaction mechanism of SOx, where sarcosine is enzymatically oxidized to release electrons, while zinc ions reversibly migrate within the electrode material to maintain charge balance. Among the considered adsorption sites, the configuration with the highest total energy, as shown in the Figure S8, illustrates the atomic arrangement of CuSe and the relative positions of Cu and Se atoms. Figure 2F shows the charge–discharge profiles of the CuSe cathode at 1.0 A g^−1^, delivering initial discharge and charge capacities of 315 and 328 mAh g^−1^ with a coulombic efficiency of 96%. The capacity remains nearly unchanged in subsequent cycles, indicating highly reversible Zn^2+^ storage behavior. Figure 2G shows the ratio performance of the prepared CuSe material. The stable discharge capacities observed at 356, 340, 331, 318, 308, 298, and 276 mAh g^−1^ at current densities of 0.1–5.0 A g^−1^, and the capacity retention rate is 78%. Moreover, the reversible capacity quickly recovers to about 300 mAh g^−1^ when the current density is adjusted to 0.1 A g^−1^, indicating that the prepared multidimensional CuSe cathode material has good magnification tolerance and cyclic stability. The calculated diffusion coefficient of the CuSe material is mainly stable in the range of 10^−11^–10^−12^ cm^2^ s^−1^, which is consistent with the charge–discharge results, confirming that the material has excellent discharge behavior (Figure 2H). As shown in Figure 2I, the long‐cycle performance of CuSe was tested at 1 A g^−1^, and the results showed that the capacity retention rate was 80% after 200 cycles, indicating that the CuSe material has excellent long‐cycle durability.

### 3.4 Sarcosine‐Related Bioinformatics Analysis

**FIGURE 3:**
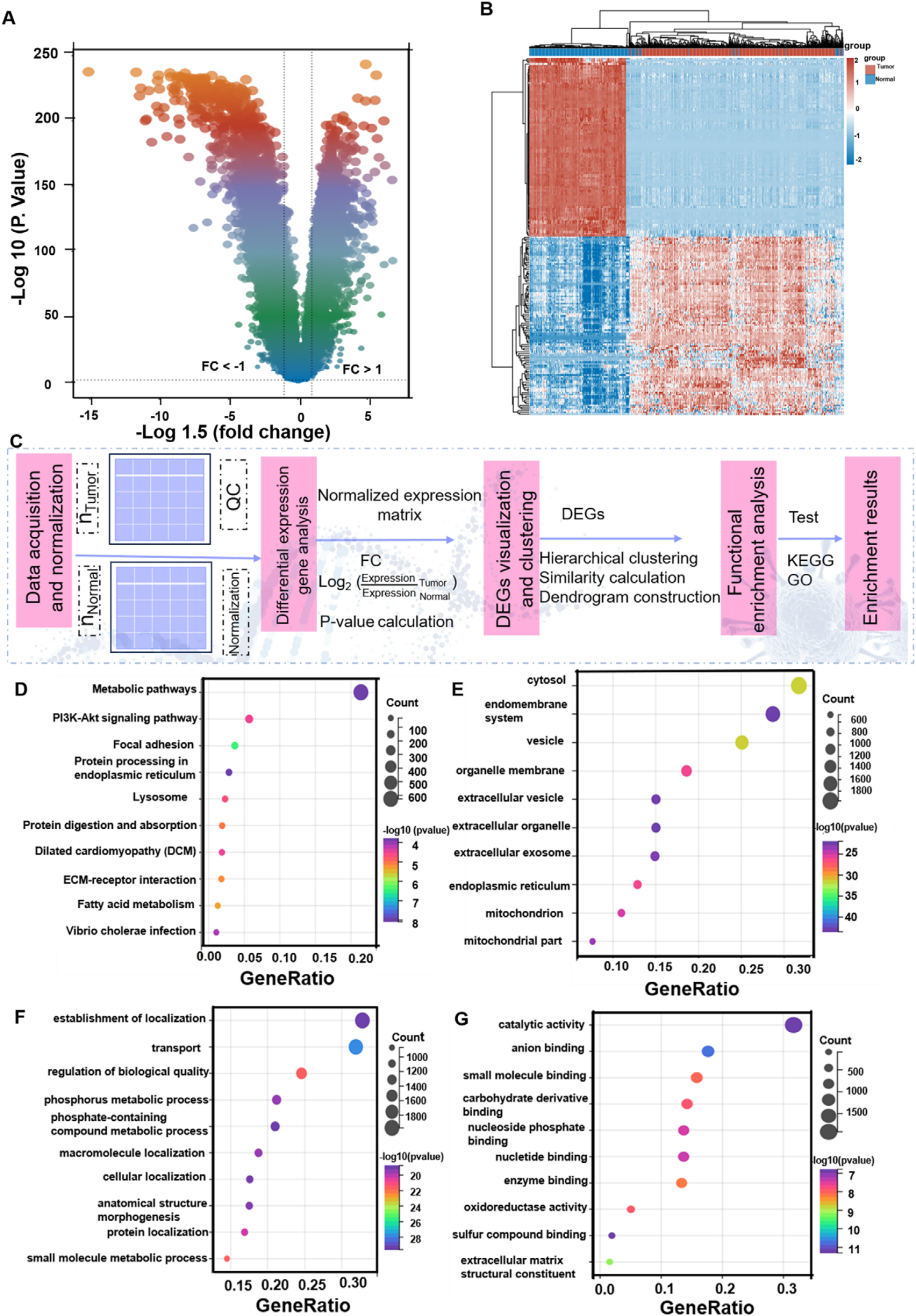
(A) Differential gene expression between prostate cancer and normal tissues, (B) Heatmap of differentially expressed genes, (C) Bioinformatics workflow for differential expression and functional enrichment analysis, (D) KEGG enrichment analysis of differentially expressed genes, (E) GO cellular component enrichment, (F) GO biological process enrichment, (G) GO molecular function enrichment.

To explore gene expression patterns associated with sarcosine‐related alterations in prostate cancer, a series of bioinformatics analyses were performed. Differential expression analysis (Figure 3A) identified multiple significantly upregulated and downregulated genes between prostate cancer and normal tissues. The heatmap with hierarchical clustering (Figure 3B) shows that these differentially expressed genes distinguish tumor samples from normal samples based on their expression profiles. The overall analytical workflow is summarized in Figure 3C. KEGG pathway enrichment analysis (Figure 3D) indicates that the differentially expressed genes are enriched in pathways related to metabolism and several signaling processes. GO cellular component analysis (Figure 3E) shows that these genes are mainly associated with intracellular structures such as organelles and membrane‐related components. GO molecular function analysis (Figure 3F) reveals enrichment in functions including small‐molecule binding and enzymatic activity. GO biological process analysis (Figure 3G) suggests involvement in biological processes such as substance transport and metabolic regulation. Overall, Figure 3A–G summarize the differential expression patterns and functional enrichment results of sarcosine‐associated genes in prostate cancer.

### 3.5 Sensor Performance

**FIGURE 4:**
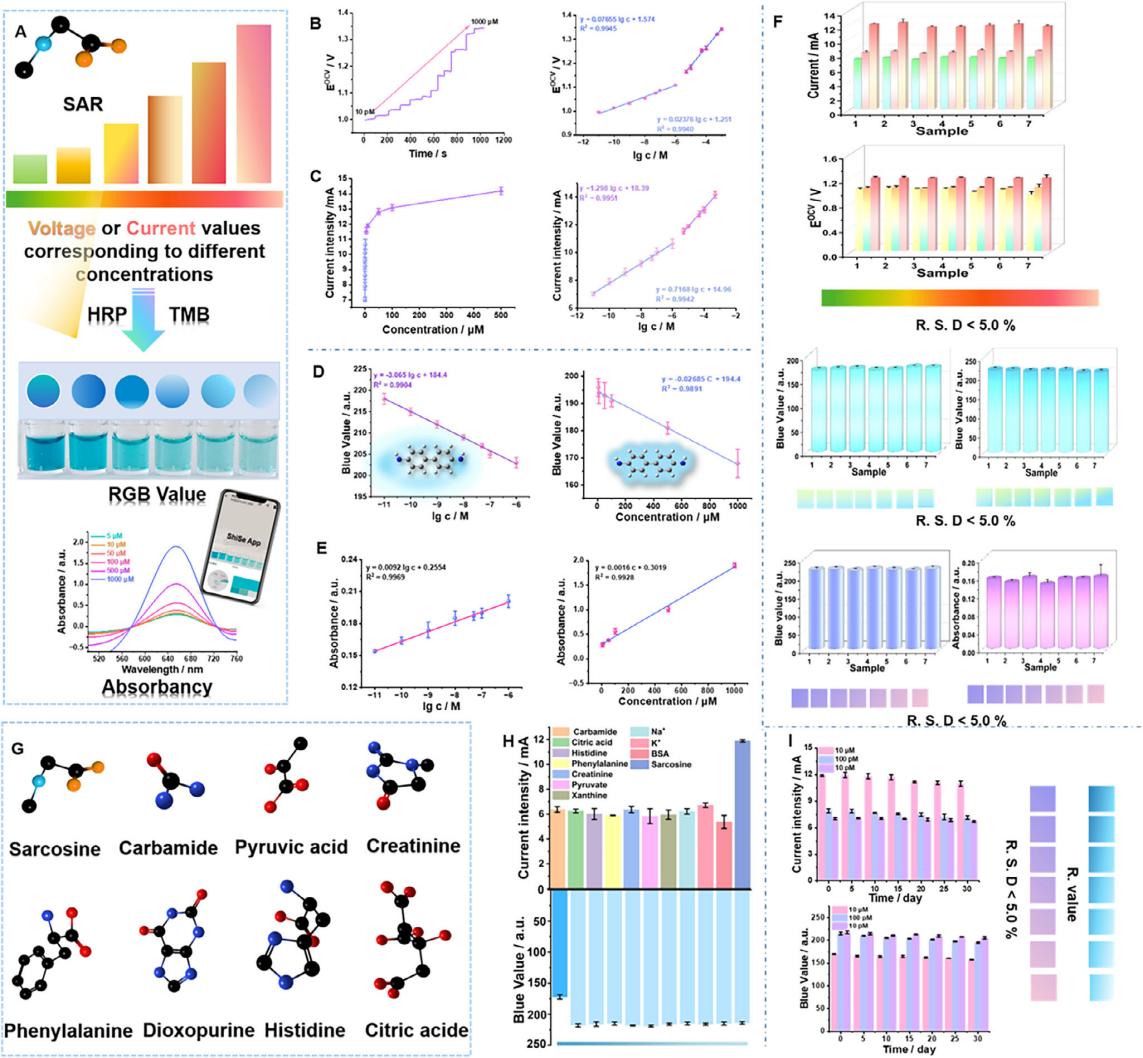
(A) Schematic illustration of the dual‐mode detection mechanism, (B) Relationship between E^OCV^ and SAR concentration, (C) Correlation between transient current and SAR concentration, (D,E) Optical detection mode showing RGB/absorbance versus SAR concentration relationships, all measured in PBS buffer containing 10 µm Zn^2+^ with varying concentrations of SAR, (F) Stability comparison of four testing methods, (G) Structural analysis diagram of interference experiments, (H) Specificity data under dual detection modes, (I) System stability tests in both electrochemical and colorimetric modes.

SEM observation revealed distinct morphological changes in the carbon cloth after coating with the electrode material (Figure S9). The coating rendered the surface noticeably rougher, with the active material uniformly distributed along the fiber network. This uniform coverage increases the effective surface area and provides more electroactive sites, thereby enhancing signal transmission. Such morphological features improve the interaction between the analyte and the electrode surface, enabling stable and sensitive sensor output. Figure 4A illustrates the dual‐mode detection mechanism of the wearable diaper, which enables accurate sarcosine concentration detection through synergistic electrochemical‐optical output. In the electrochemical detection mode, the fuel concentration of the EBFC correspondingly rises as the SAR concentration increases. SAR undergoes oxidation catalyzed by SOx, H_2_O_2_ while releasing electrons. These electrons are transferred through the electrode to the external circuit, leading to a significant enhancement in the open‐circuit voltage (E^OCV^) or short‐circuit current of the battery system. The signal intensity exhibits a positive correlation with the sarcosine concentration. Meanwhile, the H_2_O_2_ produced by the SAR oxidation reaction further triggers the optical detection mode. In the presence of horseradish peroxidase (HRP), H_2_O_2_ oxidizes the colorless substrate 3,3',5,5'‐tetramethylbenzidine (TMB), generating blue oxidized TMB (oxTMB), which induces a distinct color change in the system. The reactions can be represented as follows:

Sarcosine+O2+H2O→Glycine+Formaldehyde+H2O2

$$HRP+H2O2\to \;HRP\left(ox\right),\;HRP\left(ox\right)+TMB\;\to \;oxTMB$$

The chromogenic intensity of this electrochromic reaction is directly correlated with the H_2_O_2_ concentration, thereby establishing a quantitative relationship with the initial sarcosine concentration. The color change can be quantitatively analyzed via smartphone RGB analysis or UV–vis spectrophotometry. This dual‐mode design provides highly sensitive quantitative results through electrochemical signals and offers intuitive visual readouts via optical signals. The mutual verification between these two modes significantly enhances detection reliability, making it particularly suitable for rapid screening by non‐professional users in home settings. In this system, urinary Zn^2+^ (10 µm) serves as the electrolyte for the ZIB, which functions as an auxiliary and reversible power unit to stabilize and amplify the output signal rather than as a sensing element. Owing to the reversible Zn^2+^ insertion/extraction mechanism, normal fluctuations in Zn^2+^ concentration have negligible impact on short‐term battery operation. Figure 4B,C shows the sensitivity response curves of E^OCV^ and transient current in the electrochemical detection mode. As the target analyte concentration increases, both E^OCV^ and transient current exhibit progressive rises, demonstrating strong linear correlations (R^2^ > 0.99) over specific concentration ranges. For E^OCV^, the linear range is 10 pm–1000 µm, with calibration equations of y = 0.02376 lg c + 1.251 (R^2^ = 0.9940) and y = 0.07655 lg c + 1.574 (R^2^ = 0.9945), yielding a detection limit (LOD) of 0.95 pm. For transient current, the linear range is 10 pm‐500 µm, with calibration equations of y = 0.7168 lg c + 14.96, R^2^ = 0.9942) and y = 1.298 lg c + 18.39, R^2^ = 0.9951), with a corresponding LOD of 0.85 pm. These results indicate that both E^OCV^ and transient current responses are highly sensitive and suitable for quantitative detection of the analyte. Notably, the ultralow LOD of 0.85 pm underscores the exceptional analytical sensitivity of our sensing strategy, demonstrating its feasibility and providing a solid foundation for integration into wearable platforms. Figure 4D illustrates the quantitative capability of the colorimetric mode. The results indicate that increasing SAR concentration leads to a linear change in the blue value of the blue oxTMB product at 652 nm, which fits the equations y = −3.065 lg c + 184.4 and y = −0.02685 c + 194.4 (LOD = 1.14 pm). Figures 4E and S10 employ UV–vis spectrophotometry for cross‐verification to validate the reliability of optical detection. The calibration curve obtained at the 652 nm characteristic absorption peak closely aligns with grayscale analysis results, confirming the robust quantitative performance. Figures 4F and S11 shows the reproducibility of the dual‐mode system, with RSDs below 5% across different measurement modes. As shown in Figure 4G,H, control experiments with common interferents (e.g., creatinine, and amino acids, 10 mm each) demonstrated that only the target analyte sarcosine (SAR, 10 µm) induced significant electrochemical and colorimetric responses, confirming the high specificity of the enzymatic cascade reaction. The sensing system was operated under buffered near‐neutral conditions (pH 7.4), thereby minimizing pH‐related interference during urine analysis. Finally, system stability was evaluated at three concentrations in both electrochemical and colorimetric modes (Figure 4I). The modified electrodes were stored at 4°C under dry conditions in airtight packaging to minimize moisture exposure and enzyme degradation. Stability measurements were conducted at 5‐day intervals using electrodes prepared under identical modification conditions. The sensor retained over 90% of its initial performance after 30 days. Compared with reported sarcosine sensors (Table S1), this platform shows comparable or superior sensitivity, simpler operation, and better suitability for POCT.

### 3.6 Machine Learning Data Processing and Actual Sample Analysis

**FIGURE 5:**
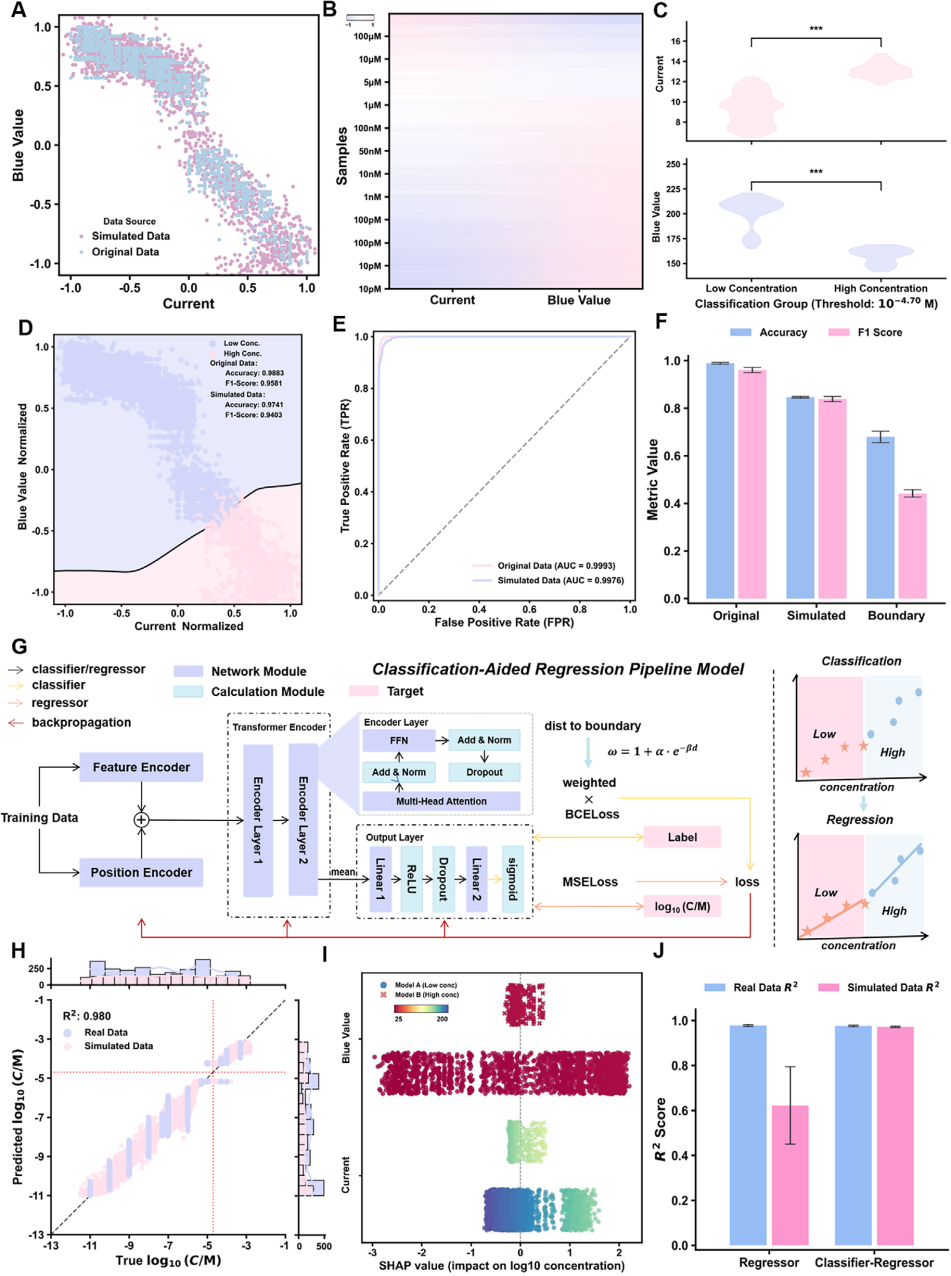
(A) Experimental and simulated data distribution. A total of 240 independently prepared samples (∼20 per concentration) were measured in triplicate after signal stabilization and averaged for model training, (B) Dual‐mode signal distribution at different concentrations, (C) Statistical comparison of low and high concentrations, (D) Classification Accuracy and *F*1‐score, (E) ROC curves and AUC values, (F) Classification of boundary samples, (G) Workflow of the classification‐regression model, (H) Predicted vs. true values after piecewise regression, (I) SHAP feature importance analysis, (J) Regression performance on real and simulated data.

Since the experimentally obtained bimodal data were distributed discretely along the concentration dimension, continuous simulated data were constructed based on the statistical distribution characteristics of the original dataset to evaluate the stability and generalization of the model in continuous space. All model training and parameter optimization were performed exclusively using experimentally acquired real data, while the simulated data were introduced only for post hoc validation and continuity analysis. As shown in Figure 5A, the original and simulated data exhibit highly consistent distributions in the 2D space of current and blue value, indicating that the simulated data effectively reproduce the overall characteristics of the experimental data. As shown in Figure 5B, samples at different concentrations display a clear gradient distribution in bimodal signals, with good separability between low‐ and high‐concentration regions. Based on this feature distribution, Figure 5C shows that both current and blue value exhibit extremely significant differences between low‐ and high‐concentration groups (*p* < 0.001), validating the rationality of the classification threshold. The dataset was divided into training and test sets using stratified random sampling prior to model development, ensuring balanced concentration distributions while preventing information leakage. Furthermore, as shown in Figure 5D, the classification model achieved high Accuracy and *F*1‐score for both the original and simulated datasets in the normalized feature space. The corresponding ROC curves (Figure 5E) shows that the AUC values are close to 1, indicating excellent discriminative ability and generalization performance. As shown in Figure 5F, the classification performance of boundary samples is notably degraded compared with that of original and simulated samples, suggesting that uncertainty near the threshold is the primary factor affecting classification stability. Figure 5G schematically illustrates the overall machine‐learning workflow consisting of dual‐modal feature input, classification, and segment‐wise regression for quantitative prediction, providing a methodological framework for the subsequent classifier–regressor integrated analysis. After introducing piecewise regression, Figure 5H shows a strong linear correlation between the predicted values and the true lg(C/M) values (R^2^ = 0.980), demonstrating a substantial improvement in quantitative prediction accuracy. The SHAP analysis (Figure 5I) reveals that the contribution weights of current and blue value differ across concentration ranges, reflecting the complementarity of the bimodal signals. In a broader clinical context, inter‐donor variability could be further mitigated through feature‐wise normalization. The incorporation of donor‐related metadata as auxiliary covariates would enable adaptive calibration without altering the core sensing mechanism. Finally, as shown in Figure 5J, the “classification‐regression” joint model significantly improves the regression fitting performance of the simulated data, making it comparable to that of the real data, which indicates that this strategy effectively mitigates the adverse impact of data distribution discrepancies on regression accuracy. To evaluate the practical applicability of the self‐powered biosensor in real samples, spike‐and‐recovery experiments were conducted. Urine samples were first buffered to pH 7.4 using Tris‐HCl and then centrifuged 3–5 times at 4°C and 4000 rpm for 5 min to reduce matrix interference. The resulting supernatant was diluted with PBS solution (pH = 7.4) and subsequently spiked with different concentrations of sarcosine. As shown in Figure S12A–D, the recovery rates of the sensor ranged from 83.68% to 125.88%, with RSDs between 2.65% and 7.95%. These results demonstrate that the proposed method is suitable for the detection of analytes in real samples.

### 3.7 Wearable Chip Testing

**FIGURE 6:**
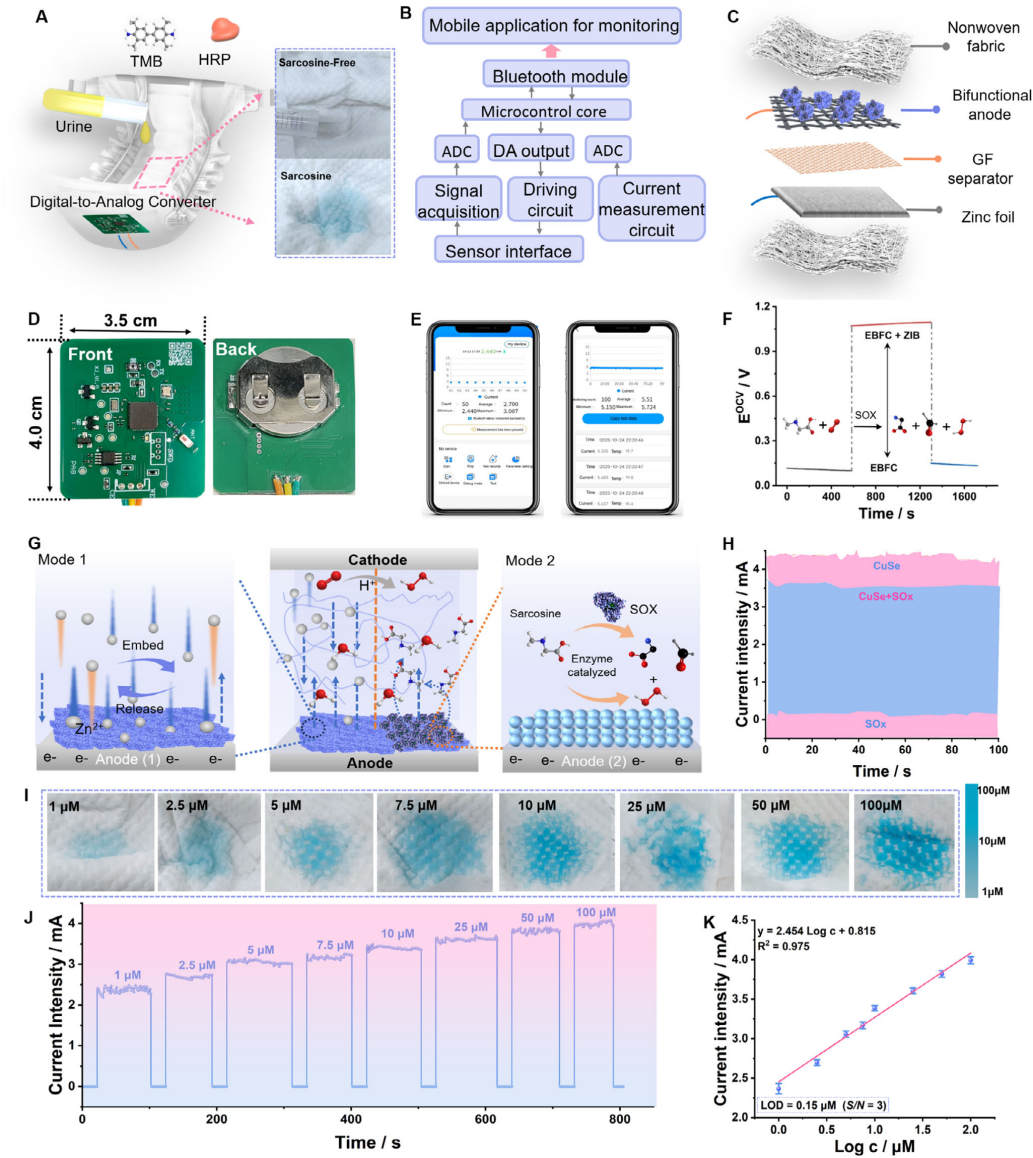
(A) Diaper‐integrated electrochemical sensor, (B) Schematic of the wireless module (ADC, analog‐to‐digital converter; DAC, digital‐to‐analog converter), (C) schematics of the integrated chip‐diaper assembly, (D) Wireless transmission module, (E) Smartphone app interface, (F) E^OCV^ validation of the single‐enzyme biofuel cell and dual‐powered enzyme‐zinc battery system, (G, H) Dual‐power mechanism model and its verification, (I) Images of diapers after exposure to different sarcosine concentrations, (J, K) Diaper‐integrated electrical responses to varying sarcosine concentrations with the fitted current‐log(concentration) calibration curve.

As shown in Figures 6A and S13, the electrochemical sensor was successfully integrated into the absorbent layer of the diaper, and its flexible structure ensured stable electrochemical performance under bending and compression. To further evaluate the mechanical robustness of the device, the E^OCV^ of the pristine, stretched, and bent self‐powered sensors were compared (Figure S14), showing only negligible variation among the three states, which confirms the reliable operational stability under mechanical deformation. Short‐term continuous data acquisition enables the capture of transient temporal variations in urinary biomarkers associated with prostate cancer risk, providing more informative early‐warning insights than single‐point measurements. To achieve instantaneous detection without external devices, the system was further combined with a wireless transmission module operating at a 0.5 s sampling interval (Figure 6B–E). In this system, the ADC and DAC modules effectively convert analog signals into digital signals, providing a reliable basis for remote data transmission. It should be noted that the power unit shown in Figure 6D is solely used to supply energy for signal processing and wireless transmission and does not participate in the sensing process. The assembly module of the self‐powered sensor demonstrates the compact integration of all components, enabling the system to operate independently without the need for an external power source. To further elucidate the source of energy and the signal‐generation mechanism, the dual‐power mechanism model and its validation results (Figure 6F–H) were analyzed. The results revealed that the synergistic interaction between the biofuel cell and the auxiliary electrochemical energy significantly enhances the output current and improves detection sensitivity by approximately one order of magnitude. For testing, urine samples were first centrifuged to remove impurities and then poured onto the absorbent diaper substrate, allowing simultaneous colorimetric and electrochemical detection. The UV–vis absorption spectra of the colorimetric system under different conditions are shown in Figure S15. No obvious absorbance was observed in the control groups, whereas a pronounced absorption peak appeared only in the simultaneous presence of HRP and SOx. This result confirms that the colorimetric response is exclusively triggered by H_2_O_2_ generated through SOx‐catalyzed oxidation of sarcosine, thereby demonstrating the high specificity of the assay against nonspecific urine‐derived interference. When different concentrations of sarcosine were applied onto the diaper surface, visually distinguishable responses could be observed (Figure 6I), with concentration‐dependent color or interfacial changes providing intuitive cues for signal interpretation. The blue oxTMB signal could be observed or analyzed using a smartphone‐based shise app. During actual signal acquisition (Figure 6J,K), the output current exhibited a strong linear correlation with the logarithm of sarcosine concentration in urine, following y = 2.454 lg c + 0.815 (R^2^ = 0.975), with a detection limit of 0.15 µm. In the diaper‐integrated configuration, the wetted diaper layer defines a characteristic diffusion path length of approximately 1–2 mm, and the output signal reaches a quasi‐steady state within a few seconds after urine contact. This result confirms that the diaper‐integrated platform can reliably quantify sarcosine in complex urine matrices. Notably, the output signal remained stable over tens of minutes during operation, indicating adequate signal stability under urine conditions. Under real urine environments and wearable operating conditions, the limit of detection increased to 0.15 µm due to matrix interference, hindered diffusion, and signal attenuation during the absorption process. Nevertheless, this detection limit remains within the clinically relevant and acceptable concentration range of sarcosine, demonstrating the feasibility of this system for early prostate cancer detection in practical applications [30, 31].

## 4 Conclusion

In summary, this work presents a urine‐powered biosensing platform with a new operating mode. The system directly harvests the intrinsic chemical energy of urine, where a metabolite‐based biofuel cell operates synergistically with a zinc–ion battery to achieve a self‐sustaining power supply. Highly sensitive and selective sarcosine detection is realized via dual‐mode electrochemical and colorimetric readouts, enabling user‐readable signal output without external power. A dual‐modal machine‐learning framework integrating classification and segment‐wise regression further improves quantitative accuracy over a broad concentration range. After integration into a wireless diaper‐based wearable format, the platform exhibited stable operation under practical testing conditions. Validation using human urine samples was conducted through spike‐and‐recovery experiments, confirming feasibility and analytical reliability in complex biological matrices. By eliminating invasive sampling and external power sources, this strategy provides a promising proof‐of‐concept for intelligent, self‐powered point‐of‐care biosensing. Moreover, the proposed system can be readily extended to the simultaneous detection of additional urinary biomarkers (e.g., PCA3, PSMA), thereby further enhancing detection sensitivity and expanding the analytical scope for early‐stage biomarker sensing.

## Conflicts of Interest

The authors declare no conflict of interest.

## Supporting information




**Supporting File**: advs74626‐sup‐0001‐SuppMat.docx.

## Data Availability

The data that support the findings of this study are available in the supplementary material of this article.
