# Supplementary material for: Toward Prostate Cancer Early Warning with a Self‐Powered Wearable Biosensing Platform Integrated with Machine Learning
Source: Adv Sci (Weinh). 2026 Feb 27:e24234. Online ahead of print. doi: 10.1002/advs.202524234 (PMC13326029; doi:10.1002/advs.202524234)
Supplement: Supplementary file 1 — Supporting File: advs74626‐sup‐0001‐SuppMat.docx. [file ADVS-9999-e24234-s001.docx]

**Supporting Information**

**Toward prostate cancer early warning with a self-powered wearable biosensing platform integrated with machine learning**

*Jing Xu^1^, Hanxiao Chen ^3^, Qichen Yuan^1^, Liucun Yin^1^ Yifang Tao^1^, Hong Wang^1^, Yuquan Xue^1^, Delai Fu^1^, Huan Pang^2*^,* *Tie Chong^1*^, Li Xue**^1*^*
1 The Second Affiliated Hospital of Xi’an Jiaotong University, Xi’an 710004, China
2 School of Chemistry and Chemical Engineering, Yangzhou University, Yangzhou 225009, China
3 College of Chemistry and Chemical Engineering, Xinyang Normal University, Xinyang 464000, China

*Corresponding author to one of the following:
Huan Pang, Yangzhou University, Email: [huanpangchem@hotmail.com](mailto:huanpangchem@hotmail.com)

Tie Chong, The Second Affiliated Hospital of Xi'an Jiaotong University, Email: [chongtie@126.com](mailto:chongtie@126.com)

Li Xue, The Second Affiliated Hospital of Xi'an Jiaotong University, Email: xueli1979@xjtu.edu.cn

**Chemicals**

The carbon paper (CP) (WOS1009) was purchased from Sigma-Aldrich. Sarcosine oxidase (≥ 30U/mg), Sarcosine (C_3_H_7_NO_2_, 99%), Creatinine (C_4_H_7_N_3_O, 99%), Xanthine (C_5_H_4_N_4_O_2_, 98%), Pyruvic acid (C_3_H_4_O_3_, 98%), Citric acid (C_6_H_8_O_7_, ≥ 99.5%), Histidine (C_6_H_9_N_3_O_2_, 99%), Phenylalanine (C_9_H_11_NO_2_, 98%), Carbamide (CH_4_N_2_O, 99%) and Polyvinylidene Fluoride were purchased from Macklin Biochemical Technology Co., Ltd., Shanghai, China. CuSO_4_·5H_2_O (≥ 99%), NaOH (≥ 97%), selenium (≥ 99%), sodium chloride (AR, 99.5%), ascorbic acid (≥ 99%) were purchased from McLean Biochemical Reagent Co., Ltd. 1-ethyl-3-(3’-dimethylaminopropyl) carbodiimide (EDC, ≥ 98%), N hydroxysuccinimide (NHS, ≥ 98%), monosodium dihydrogen phosphate (NaH_2_PO_4_, ≥ 99%), ethanol (C_2_H_6_OH, ≥ 99%), and disodium hydrogen phosphate (Na_2_HPO_4_, ≥ 99%) were obtained from Sinopharm Chemical Reagent Co. Ltd (Shanghai, China).

The buffer solutions for the experiment were as follows: phosphate buffered solutions (PBS) consisted of 0.1 M NaCl, 0.1 M Na_2_HPO_4_, and 0.1 M NaH_2_PO_4_ (pH 7.4).

**Synthesis of CuSe**

Dissolve specific amounts of anhydrous copper sulfate (CuSO_4_), sodium citrate (Na_3_C_6_H_5_O_7_), sodium hydroxide (NaOH), and ascorbic acid (C_6_H_8_O_6_) in deionized water. After centrifugation, washing, and drying, a cubic-structured Cu_2_O precursor can be obtained. A small quantity of this Cu_2_O precursor is taken, and selenium powder is uniformly dispersed in water via ultrasonication. A measured amount of sodium borohydride (NaBH_4_) is then added, leading to a vigorous reaction. Hydrochloric acid (HCl) is introduced into the mixture, followed by continuous stirring at 80 °C for 2 h. Finally, the target product CuSe nanomaterial is obtained by alternate centrifugation washing with ethanol and deionized water, and subsequent drying.

**Experimental Parameters**

The morphology information were analyzed by using scanning electron microscopy (SEM, S 4800). Cyclic voltammetry (CV) and AC impedance (EIS) tests were performed on a VMP3 multichannel electrochemical workstation. The battery cycle performance and GITT tests were tested on the LAND CT2001A Blue Battery Test System. The calculations were performed by density functional theory (DFT) with the Vienna Ab initio Simulation Package code. The geometry optimizations and total energy calculations were carried out using the frozen-core projector-augmented-wave method and the generalized gradient approximation for exchange correlation. The kinetic energy cutoff with a plane wave set to be 450 eV. The constructed geometric structure was relaxed until the total energy and force per atom were less than 10^−5^ eV and −0.01 eV*Å−1. The first Brillouin zone integrations were performed using 5 × 5 × 1 k-points (Monkhorst-Pack method). And the long-range dispersion correction scheme of Grimme (DFT-D3) was employed.

**
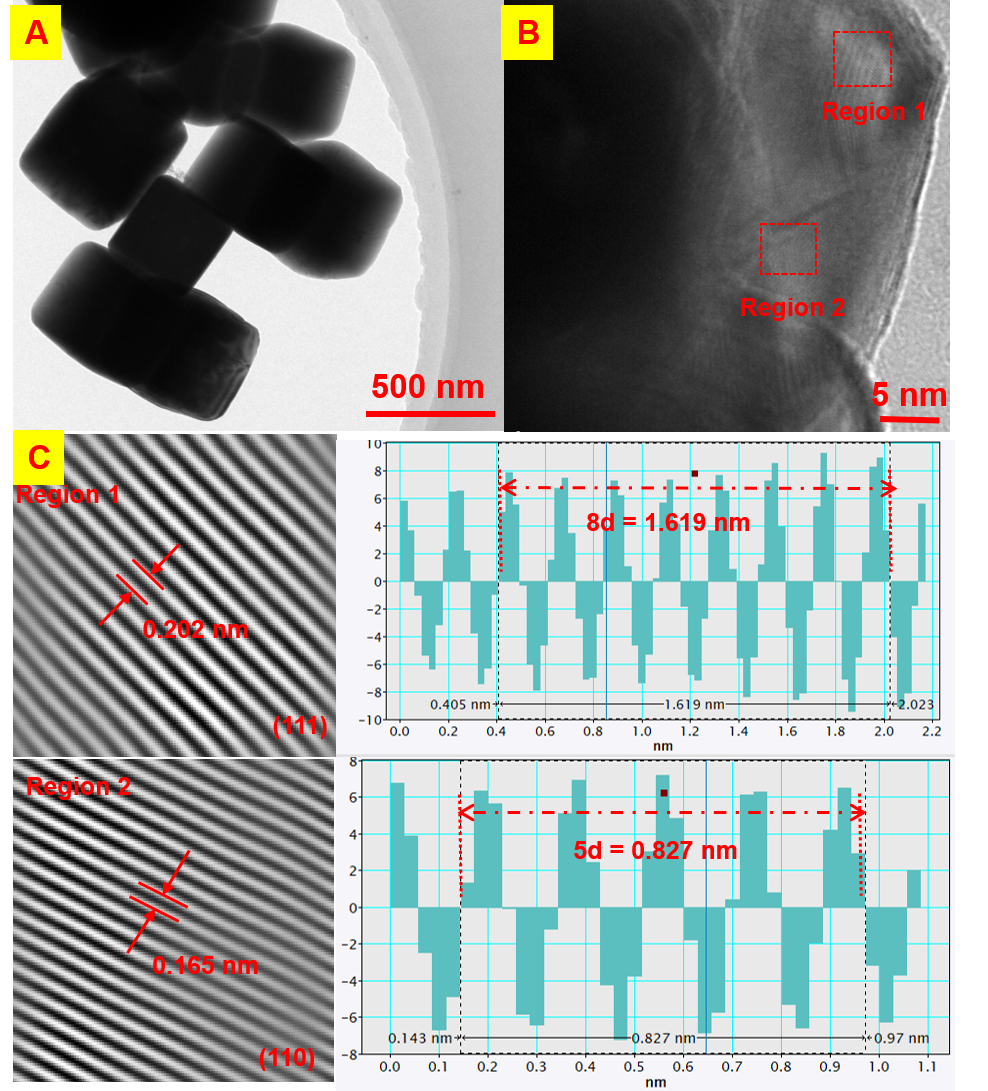
Figure S1**. (A) Transmission electron microscopy (TEM) image of Cu_2_O particles, showing their overall morphology and size distribution. (B, C) High-magnification TEM images of Cu_2_O, providing detailed visualization of the lattice fringes and nanoscale structural features.


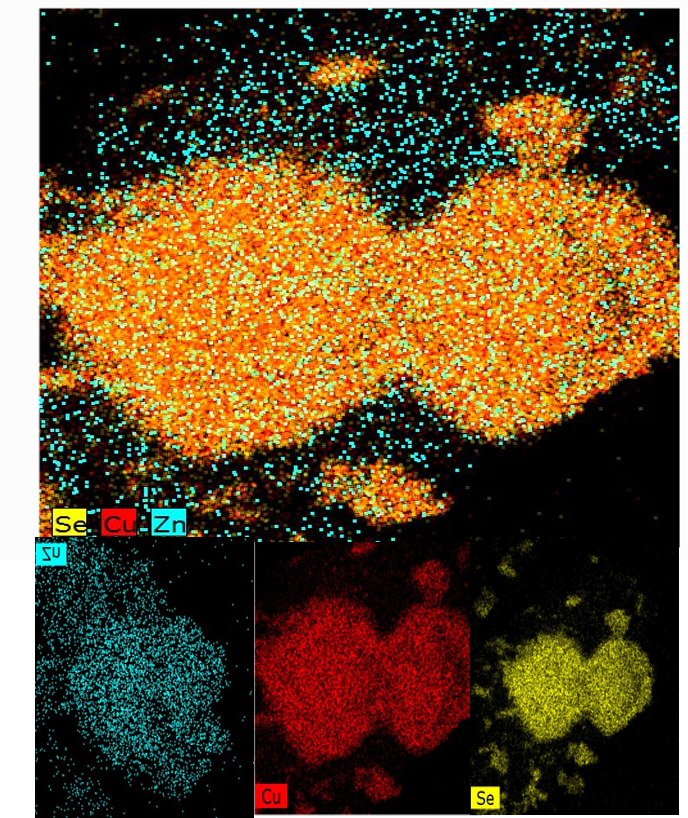
**Figure S2.** mapping images of CuSe after.


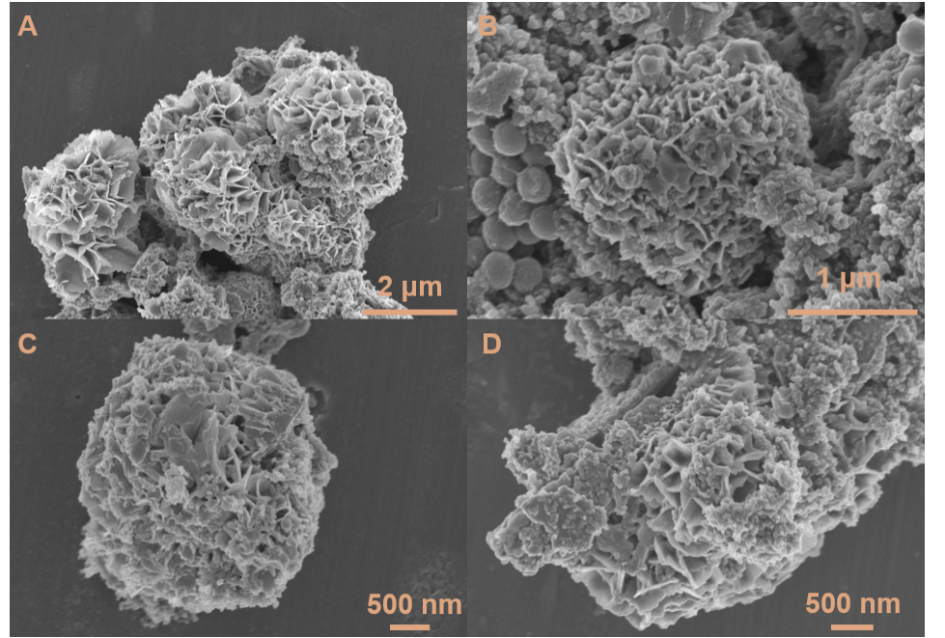
**Figure S3.** (A-D) SEM images of CuSe after electrochemical cycling, illustrating the surface morphology, structural integrity, and possible changes in particle aggregation or roughness induced by repeated charge–discharge processes.

**
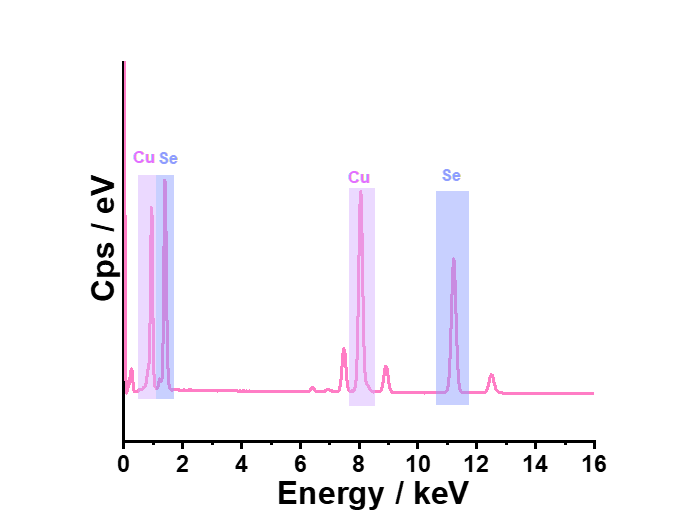
Figure S4.** EDS spectrum of CuSe.


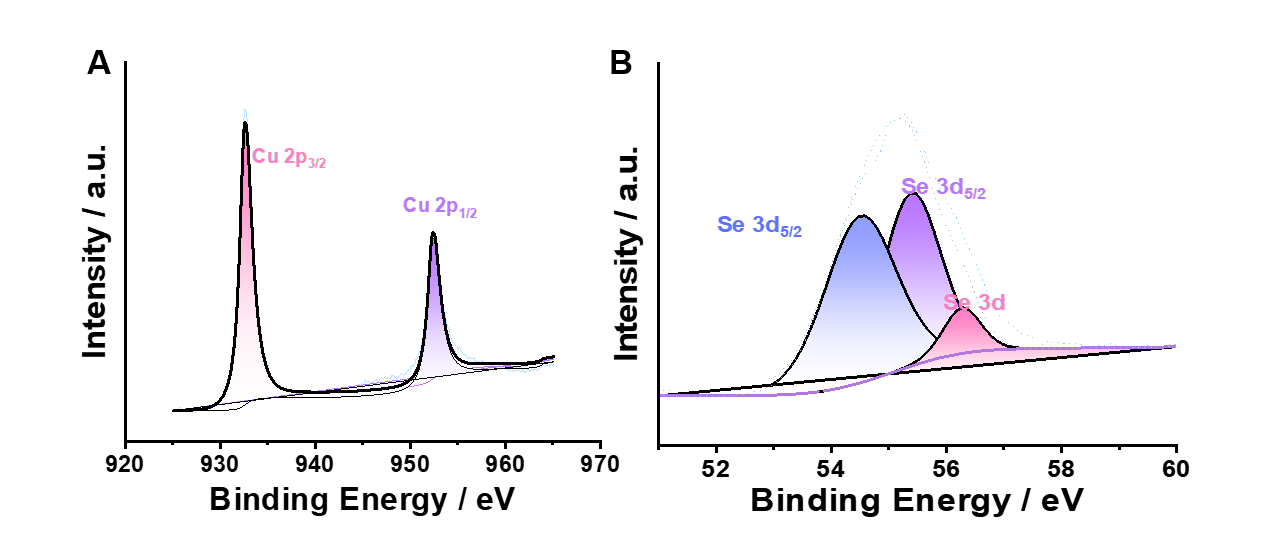


**Figure S5.** XPS spectra of CuSe: high-resolution Cu 2p and Se 3d spectra (A, B)


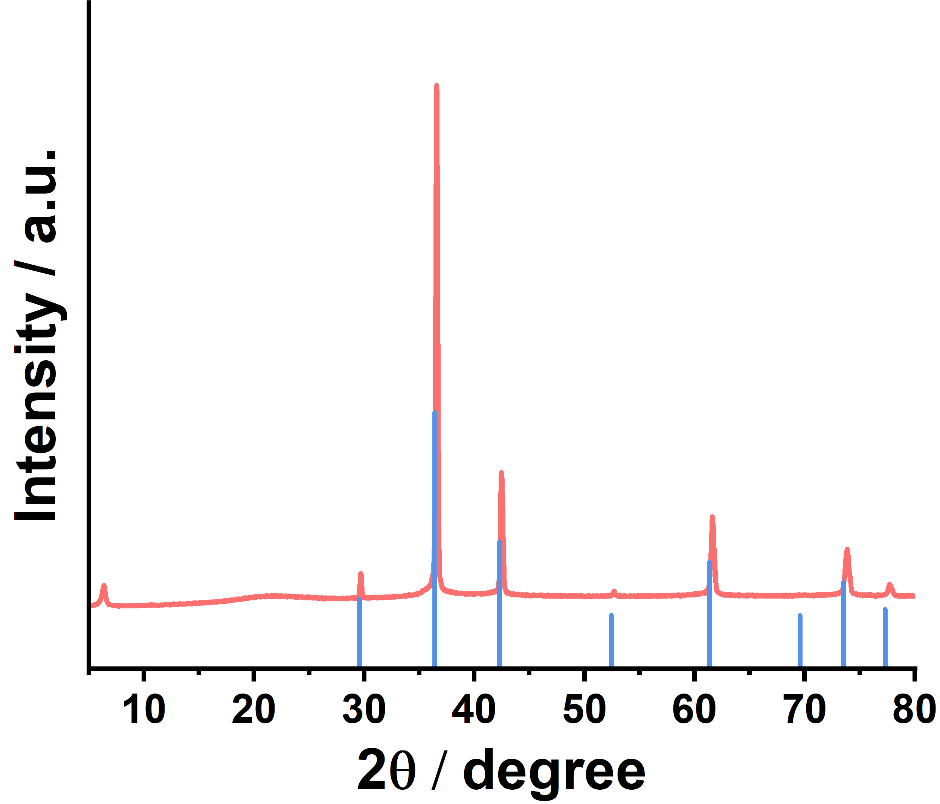
**Figure S6.** XRD pattern of Cu_2_O (PDF#05-0667).


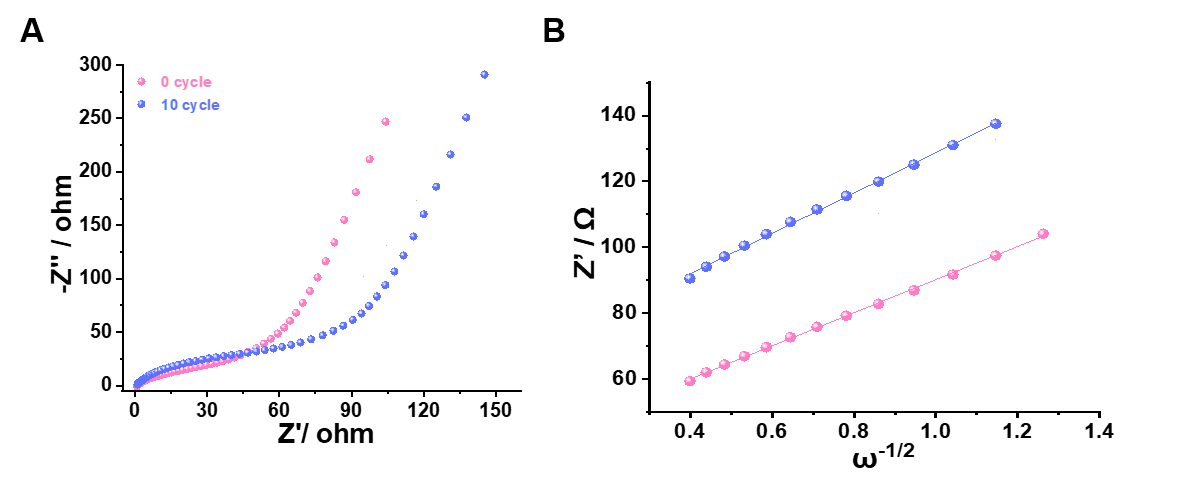


**Figure S7.** (A) Impedance before and after 10^th^ cycling, (B) Relation between the real part impedance and ω^−1/2^.


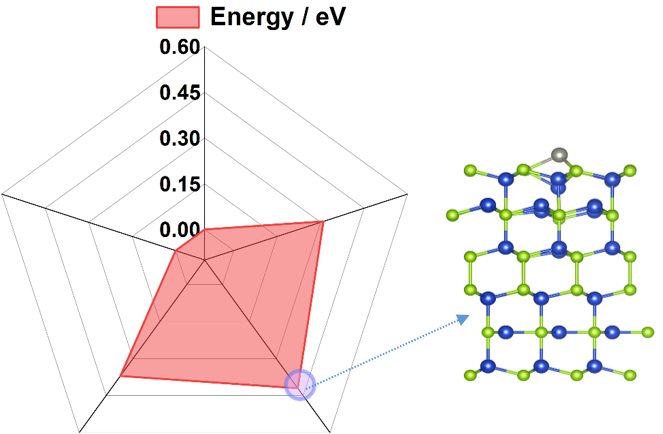


**Figure S8.** Energy profile of CuSe under different adsorption/configuration states

**
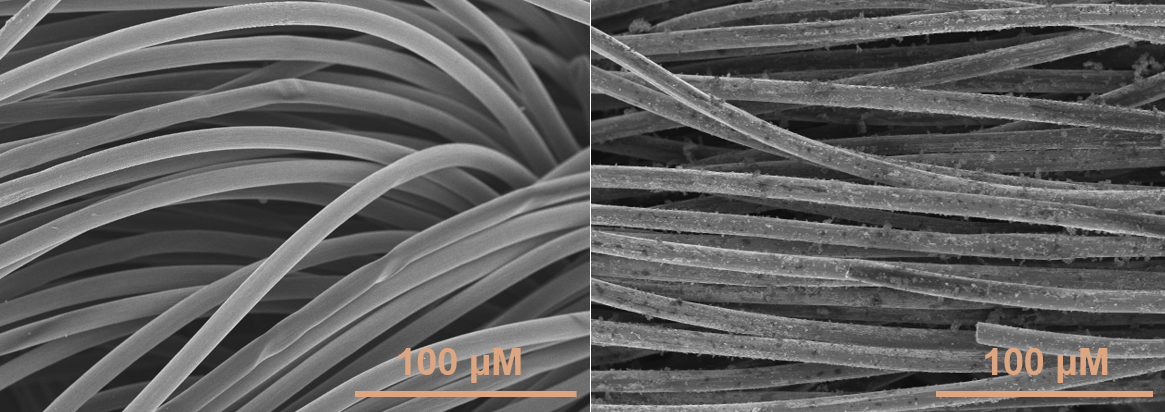
****Figure S9.** SEM images of carbon cloth before and after electrode material coating. (A) Pristine carbon cloth, showing smooth fiber bundles; (B) Carbon cloth after coating, with electrode material uniformly deposited, increasing surface roughness and providing abundant electroactive sites.


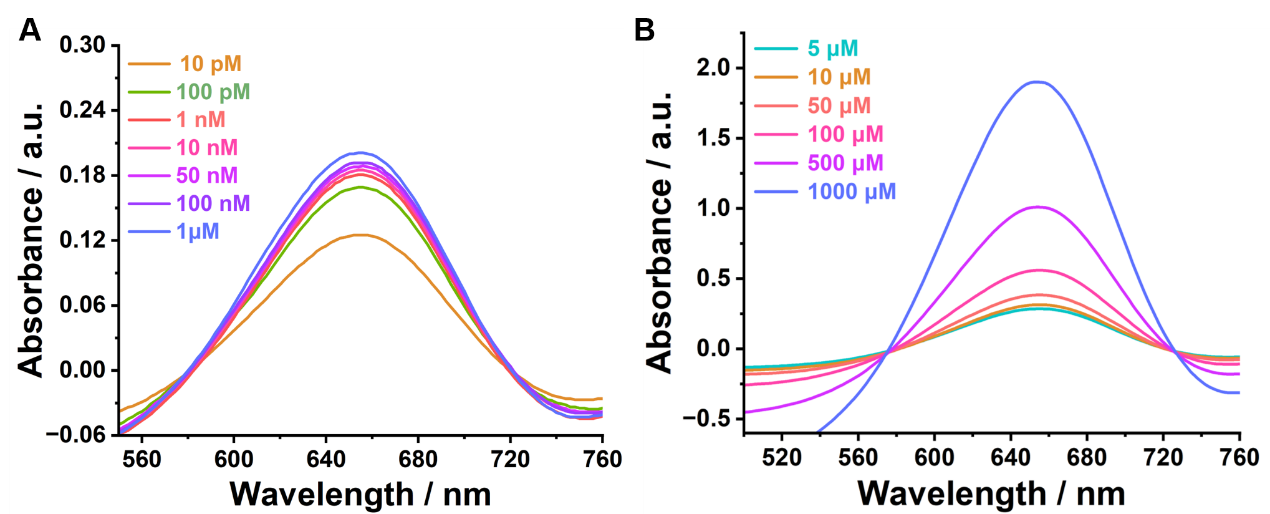


**Figure S10.** UV-vis spectra showing the relationship between absorbance and bacterial concentration determined by the UV-visible spectrophotometry method.


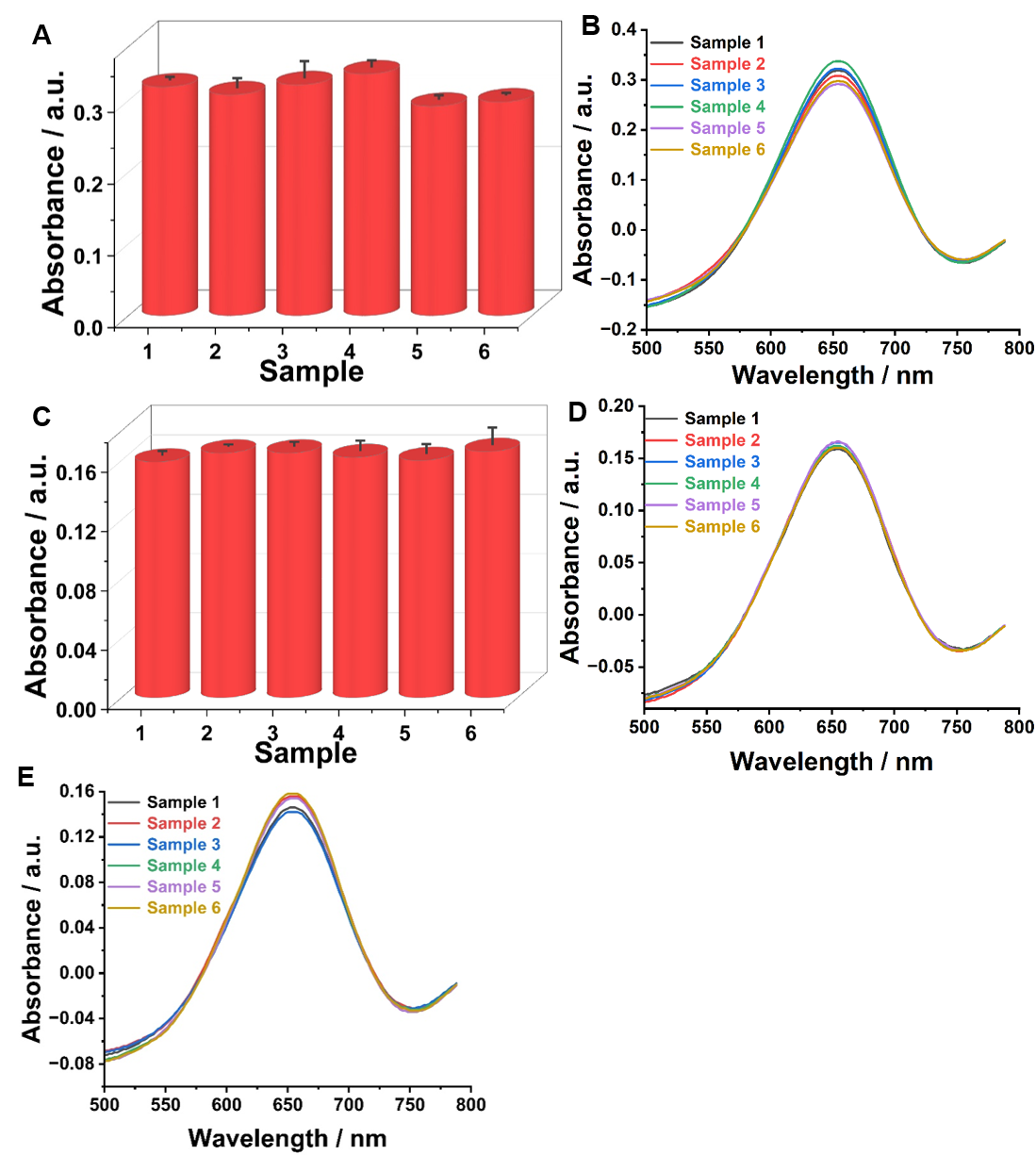


**Figure S11**. In colorimetric mode, the UV-vis spectra were used to evaluate the sensor’s reproducibility for different concentrations of sarcosine: 10 μM (A, B), 100 pM (C, D), and 10 pM (E).


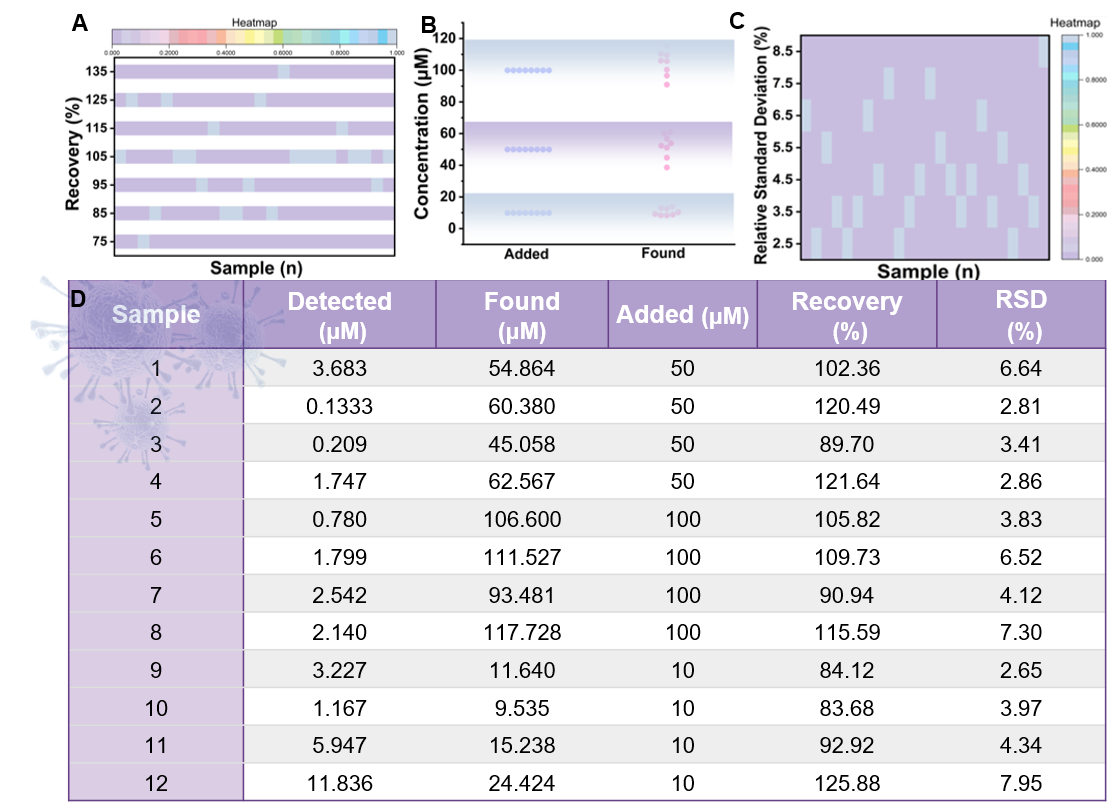
**Figure S12.** (A-D) Analysis results of human urine samples, the recovery rates of the sensor ranged from 83.68% to 125.88%, with RSDs between 2.65% and 7.95%.


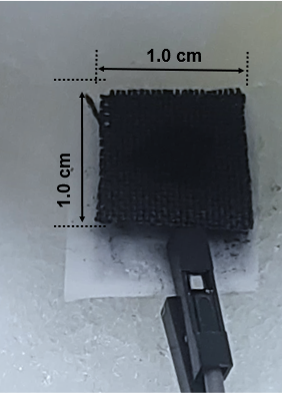


**Figure S13.** Photograph of the carbon cloth electrode with an effective area of 1.0 × 1.0 cm^2^, identical to that of the zinc foil anode used in the device.


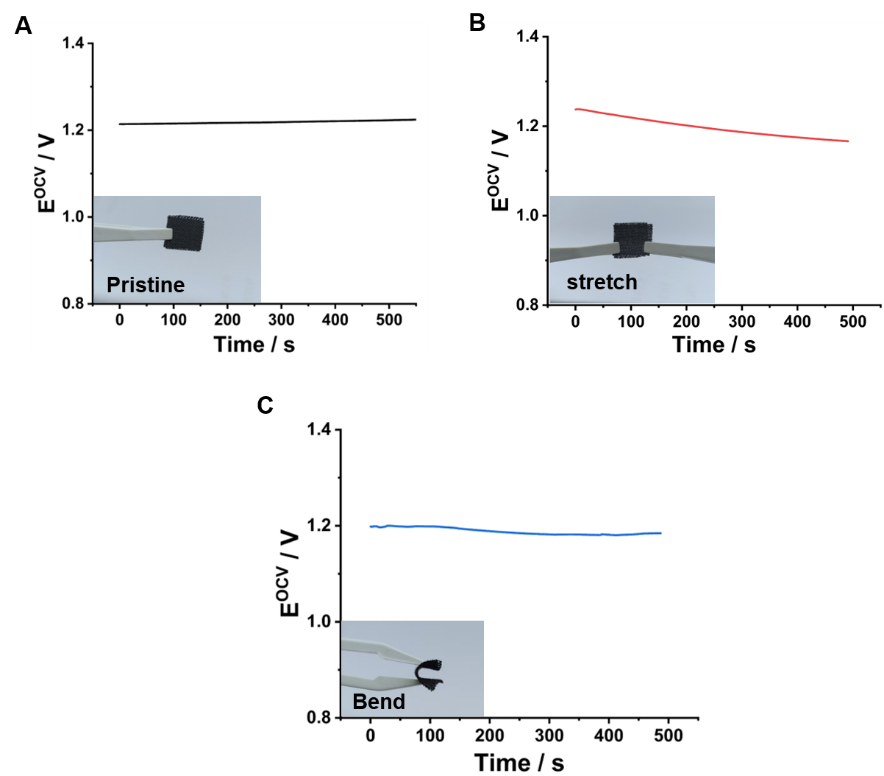
**Figure S14.** EOCV of the self-powered sensor in (A) the pristine state, (B) under 10 uniaxial tensile strain, and (C) under bending deformation.


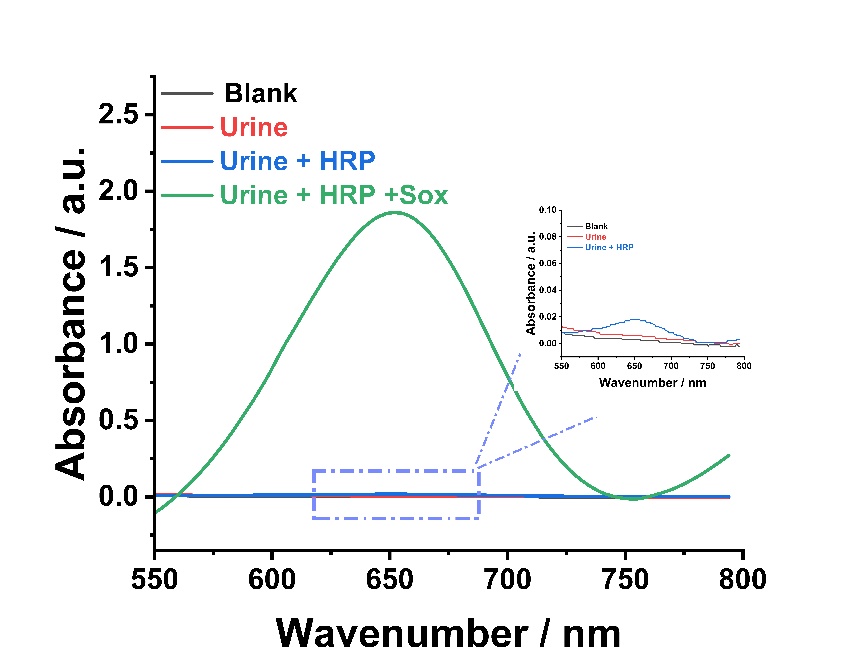
**Figure S15.** The UV-vis absorption spectra of the colorimetric system under different conditions are shown as follows: the blank sample, the sample containing urine only, the sample containing urine with HRP, and the sample containing urine with both HRP and SOx.

**Table S1**. The comparison of methods for the detection of sarcosine.

| **Method** | **Linear** **range** | **Limit of detection** | **Ref.** |
| --- | --- | --- | --- |
| Aptamer-based assay | 5 pM-50 μM/5 pM-500 μM | 0.5 pM | [1] |
| Colorimetric assay | 2 μM - 500 μM | 1.32 μM | [2] |
| Colorimetric assay | 1μM -400 μM | 0.324 μM | [3] |
| PB-DMFC/biosensor | 0.1μM - 1000 μM | 0.066 μM | [4] |
| Self-powered Sensor | 10 pM-500 μM  10 pM-1000μM | 0.85 pM  0.95 pM | This work |

**References**

[1] C. Özyurt, Z. Ç. Canbay, E. Dinçkaya, S. Evran, A highly sensitive DNA aptamer-based fluorescence assay for sarcosine detection down to picomolar levels. International Journal of Biological Macromolecules. 2019, 129, 91-97.

[2] P. Liu, Y. Liu, Z. Gai, F. Yang, Y. Yang, Highly specific colorimetric detection of sarcosine using surface molecular imprinted Zn/Ce-ZIF, Journal of Colloid and Interface Science. 2025, 681, 239-249.

[3] S. Li, K. Ge, X. Huo, K. Yang, X. Wang, Y. Yang, 2D Fe/Co-MOF/SOX cascade reactors for fast noninvasive detection of sarcosine level in prostate cancer urine, Journal of Colloid and Interface Science. 2025, 679(Part B): 401-411.

[4] L. P. T. Carneiro, A. M. F. R. Pinto, M. G. F. Sales, Development of an innovative flexible paper-based methanol fuel cell (PB-DMFC) sensing platform-pplication to sarcosine detection, Chemical Engineering Journal. 2023, 452(PartA): 139563.
